# Association between dietary omega-3 intake and coronary heart disease among American adults: The NHANES, 1999–2018

**DOI:** 10.1371/journal.pone.0294861

**Published:** 2023-12-20

**Authors:** Mengjie Zhao, Mengli Xiao, Qin Tan, Jinjin Ji, Fang Lu

**Affiliations:** 1 China Academy of Chinese Medicine Sciences, Xiyuan Hospital, Beijing, China; 2 NMPA Key Laboratory for Clinical Research and Evaluation of Traditional Chinese Medicine, Beijing, China; 3 National Clinical Research Center for Chinese Medicine Cardiology, Beijing, China; Dynamical Business & Science Society - DBSS International SAS, COLOMBIA

## Abstract

**Background:**

Omega-3 has been extensively studied for its cardiovascular disease (CVD) benefits. However, the results of this evidence are inconsistent. Therefore, in this study, dietary omega-3 intake was investigated further in relation to coronary heart disease (CHD) risk among U.S. adults.

**Methods:**

We used data from the National Health and Nutrition Examination Survey (NHANES) database for people ages 20 years and older between 1999 and 2018 to conduct a cross-sectional survey. The Medical Condition Questionnaire (MCQ) was used to determine CHD status. We measured dietary omega-3 intake using two 24-hour dietary recall interviews. Multivariate logistic regression and subgroup analysis were used to explore the correlation between dietary omega-3 intake and CHD. The dose-response relationship between the two was analyzed with a restricted cubic spline (RCS).

**Results:**

31,184 study subjects were included, of whom 1,604 (5.14%) were patients with CHD. By quintile (Q) of dietary omega-3 intake, after adjusting for all confounding factors, compared with Q1, when total dietary omega-3, alpha-linolenic acid (ALA), docosapentaenoic acid (DPA), eicosatetraenoic acid (ETA), eicosapentaenoic acid (EPA), and docosahexenoic acid (DHA) intake reached Q5, the odds ratio (95% confidence interval, CI) of CHD were 0.76 (0.60, 0.96), 0.73 (0.57, 0.94), 0.70 (0.54, 0.92), 0.66 (0.50, 0.85), 0.84 (0.69, 1.02), and 0.83 (0.64, 1.07), respectively, while EPA and DHA were not significantly associated with the disease (Trend p > 0.05). Intake of omega-3 and CHD were linearly related (*P* for nonlinear = 0.603). No significant interactions were found within subgroups except for the age group (P for interaction = 0.001). Sensitivity analysis and multivariate logistic regression results are generally in agreement.

**Conclusions:**

Total dietary omega-3, ALA, DPA, and ETA intake were negatively associated with CHD risk. In contrast, EPA and DHA had no significant correlation with CHD.

## Introduction

Cardiovascular diseases have become a significant global health burden [1]. Between 1990 and 2019, the global mortality rate of CHD further increased from 106.47/100,000 to 118.10/100,000, and the proportion of deaths to total deaths increased from 12.21% to 16.17%, making CHD the leading cause of death worldwide [2]. The total number of people with CHD in the U.S. population is 8.867 million, accounting for about 2.85% of the total population. About 558,000 people die from CHD yearly, accounting for 18.93% of the total annual deaths [2]. With the widespread prevalence of unhealthy lifestyles and CHD risk factors such as hypertension, diabetes, and sleep disorders, CHD has become the "number one killer" of human health and has brought enormous health and economic pressure to individuals and society.

Nutrition plays a crucial role in cardiovascular health [3, 4]. More and more studies have begun to explore the early risk factors and potential beneficial factors affecting the incidence of CHD from people’s daily living behaviors and diets [5–8]. Numerous studies have indicated that fish, fiber, protein, and nutrients are associated with CHD and can either reduce or raise the risk [9–11]. Therefore, it seems necessary to effectively identify potential beneficial or risk factors in the diet and provide information to prevent CHD.

Recently, increasing attention has been focused on omega-3 fatty acids. As polyunsaturated fatty acids, omega-3 fatty acids mainly contain alpha-linolenic acid (ALA), eicosapentaenoic acid (EPA) and docosahexenoic acid (DHA), as well as docosapentaenoic acid (DPA). The body obtains omega-3 from fatty deep-sea fish oils, soybean oil, and other foods. There is evidence that increased intake of omega-3 (DHA and EPA) can lower the risk of CHD and cardiovascular mortality [12–14]. In addition, the Dietary Guidelines for Americans recommend an adequate intake of omega-3 (at least 250 mg of EPA and DHA daily) to reduce the risk of cardiac death due to cardiovascular disease (CVD) [15]. Some studies have shown that omega-3 (DHA and EPA) may also benefit CHD patients by reducing atherosclerotic plaque volume [16]. However, the effectiveness of omega-3 in CVD is still controversial [17]. According to a previous large meta-analysis, omega-3 (DHA and EPA) intake is not significantly associated with cardiovascular events [18]. Several recent studies had also noted a reduced risk of cardiovascular events only when EPA was used alone, with a nonsignificant effect when combined with DHA [19, 20]. The discrepancy in these results creates considerable uncertainty in the relationship between omega-3 and CVD.

Therefore, there is no consensus on nutritional recommendations for dietary omega-3 supplementation for cardiovascular protective effects [21]. In this study, we examined the relationship between dietary omega-3 intake and CHD in U.S. adults by integrating data from 1999–2018 the National Health and Nutrition Examination Survey (NHANES) to provide dietary nutrition guidance for CHD patients.

## Methods

### Study population

This study used data from the 1999–2018 NHANES database. The NHANES survey evaluates the nutritional and health status of the U.S. national population through interviews, physical examinations, and laboratories, using a complex multilevel, stratified, and clustered sampling design to draw the sample so that the survey is highly representative of the U.S. population [22, 23]. The National Center for Health Statistics (NCHS) ethics review committee reviewed and approved all protocol implementations, and informed consent forms were signed by all participants in the study [22]. This study used publicly available data from NHANES and did not require additional ethical approval or authorization [24]. This study’s definition of CHD was derived from participants’ self-reports. Respondents were assessed as having CHD based on their responses to the health questionnaire "Have you been told by a doctor or other health professional that you have CHD?" [25]. Individuals lacking data on CHD status and omega-3 intake were excluded. In addition, we excluded individuals younger than 20 years of age, pregnant, or those with missing data on covariates of interest.

### Assessment of total dietary omega-3 intake

Total dietary omega-3 intake in this study was calculated by summing the intakes of ALA (18:3n-3), DPA (22:5n-3), eicosatetraenoic acid (ETA, 20:4n-3), EPA (20:5n-3), and DHA (22:6n-3) [26]. Omega-3 component intake data were obtained from diet-related questionnaires using a 24-hour dietary review method and the food frequency questionnaire (FFQ), which follows the automated multiple-pass method (AMPM) and enables the obtained nutritional intake data to differ from the actual intake by within 10% [27]. Food and beverages consumed during the previous 24 hours were asked to be recalled during the interview. During the first dietary assessment, professionally trained visitors interviewed the subjects and collected detailed diet information in the previous 24 hours. After days 3–10, a telephone call was made to schedule the second visit. Dietary omega-3 intake was calculated as the average intake from the two 24-hour dietary review surveys, and only the results of the first survey were used if the subject did not complete both surveys.

### Covariates assessment

The following covariates were selected for this study based on previous studies on omega-3 and CHD [28–32] and clinical reality: age, gender, race/ethnicity, education, marital status, poverty income ratio (PIR), smoking status, alcohol intake, stroke, hypertension, hyperlipidemia, diabetes mellitus, use of dietary supplements, body mass index (BMI), high-density lipoprotein cholesterol (HDL-C), and total cholesterol (TC). In this study, age was categorized into three groups: samples aged 20–44 were defined as young adults, samples aged 45–64 were defined as middle-aged, and samples aged 65 and older were defined as elderly. Race was divided into non-Hispanic white, non-Hispanic black, Mexican American, and other categories. Educational attainment was classified as less than high school, high school, and above high school. Two types of marriage status were married or living with a partner and living alone. Poverty level was expressed using PIR, with smaller PIR representing higher poverty. For this study, the sample was divided into two groups, PIR < 1 and PIR ≥ 1 [33]. The sample was defined as smoking if they smoked over 100 cigarettes in their lifetime, while the rest were non-smokers. Alcohol intake was obtained from a 24-hour dietary retrospective interview. Diagnosis of preexisting conditions (stroke, hypertension, hyperlipidemia, diabetes) was obtained based on the respondents’ responses in the questionnaire on whether they were definitively diagnosed. Dietary supplement use was judged based on the patient’s response to the questionnaire on whether they were taking any dietary supplements. Using the height (H, m) and weight (W, kg) information from the physical examination, the BMI was calculated (BMI = W/H^2^, kg/m^2^). The sample data were classified according to the international BMI classification criteria recommended by WHO [34], with BMI between 18.5 and 24.9 as normal weight, 18.4 and below as low weight, 25–29.9 as overweight, and 30 and above as obesity. HDL-C levels were measured by a heparin-manganese (Mn) precipitation method, while TC levels were determined using an enzymatic assay.

### Statistical analysis

Continuous variables with normal distributions were expressed as mean ± standard deviation (SD); variables with skewed distributions were expressed as the median and interquartile range (IQR). Frequency and percentage were used to express categorical variables. To compare group differences among normally distributed continuous variables, skewed continuous variables, and categorical variables, one-way ANOVA, Kruskal-Wallis, and chi-square tests were used. Stratification and weighting in NHANES complex sampling were considered in baseline characterization and correlation analysis. In order to investigate the association between omega-3 intake and CHD, we used a multivariate logistic regression model. Model 1 was adjusted for age, sex, race/ethnicity, education, marital status, and PIR. Based on model 1, model 2 adjusted for smoking, alcohol intake, stroke, hypertension, hyperlipidemia, and diabetes. Based on model 2, model 3 is adjusted for dietary supplements, BMI, HDL-C, and TC. A subgroup analysis was conducted to determine whether age, sex, education level, marital status, PIR, and BMI altered the effects of omega-3 intake on CHD. A likelihood ratio test was used to estimate the interaction between dietary omega-3 intake and hierarchical covariates. Moreover, we examined the relationship between dietary omega-3 exposure and CHD using restricted cubic spline (RCS) with nodes representing the 5th, 35th, 65th, and 95th percentiles to eliminate the interference of extreme outliers at the 5% before and after the data. Statistical analysis was performed using Stata 16.0 after NHANES data were collated and preprocessed using R 4.1.2. Statistics were considered significant when the P < 0.05.

## Results

### Participants

**Fig 1:**
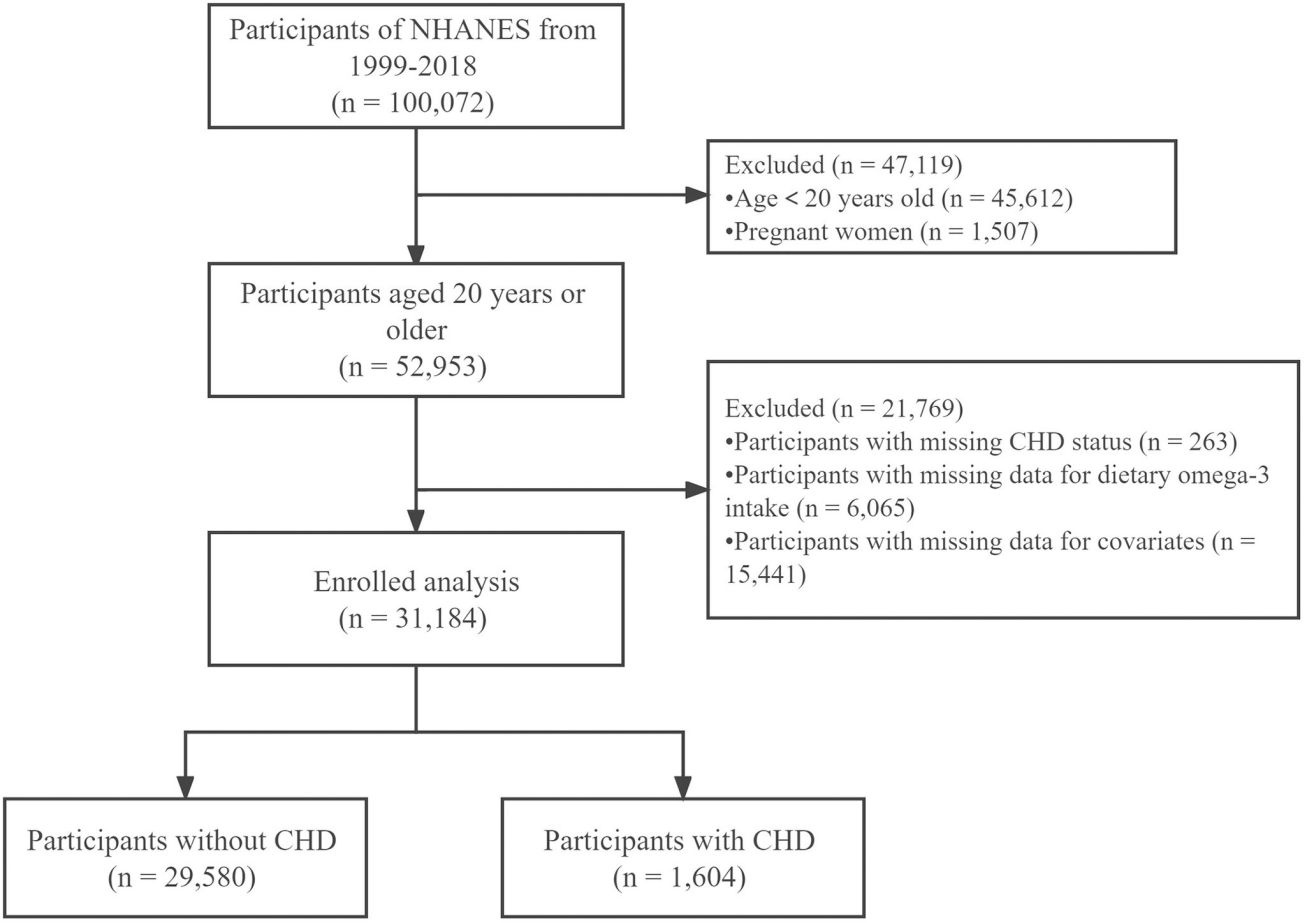
Flow chart of subject inclusion.

A total of 31,184 study participants were included in this study, of whom 1,604 (5.14%) were CHD patients and 29,580 (95.86%) were non-CHD patients (Fig 1). Participants aged under 20 years (n = 45,612), pregnant women (n = 1,507), lack of CHD status (n = 263), lack of data on dietary omega-3 intake (n = 6,065), and lack of individuals with covariates (n = 15,441) were excluded.

### Baseline characteristics of participants

Based on the CHD status of participants, Table 1 shows the baseline characteristics of these individuals. Of the 31,184 participants, 51.67% were female, 72.62% were Mexican American, and 63.65% had completed high school or higher. The participants’ median age was 49 (36.00, 61.00). It was found that CHD patients were older, more likely to be male, Mexican-American, obese, smokers, and had a lower level of education than non-CHD patients (P < 0.001). CHD patients also had a greater prevalence of stroke, hypertension, hyperlipidemia, and diabetes (P < 0.001). Patients with CHD also consumed fewer dietary supplements and alcohol than non-CHD (P < 0.001). It is noteworthy that patients with CHD consumed lower dietary omega-3 intakes than non-CHD patients (1.52 (1.03, 2.26) vs 1.62 (1.10, 2.36); P < 0.001). In addition, we analyzed the baseline characteristics of the participants according to their omega-3 intake quintiles (S1 Table). Those who consumed more omega-3 were likely to be younger, male, Mexican American, married or cohabiting, and have higher education levels and household income (P < 0.001). In addition, as omega-3 intake increased, the number of strokes, hypertension, and diabetes decreased, and TC decreased, but at the same time, alcohol intake increased, and the percentage of obese people increased (P < 0.05).

**Table 1:** Comparison of baseline characteristics of the included population.

Variables	Overall (n* = 31,184)	Non-CHD (n = 29,580)	CHD (n = 1,604)	P-value
Age (years), Median (IQR)	49.00 (36.00, 61.00)	48.00 (36.00, 60.00)	68.00 (60.00, 76.00)	< 0.001
Age (years), n (%)				< 0.001
20–44 years	11,315 (40.62)	11,267 (42.19)	48 (4.40)	
45–64 years	11,352 (39.39)	10,887 (39.65)	465 (33.54)	
≥ 65 years	8,517 (19.98)	7,426 (18.16)	1,091 (62.05)	
Sex, n (%)				< 0.001
Male	15,336 (48.33)	14,214 (47.53)	1,122 (67.00)	
Female	15,848 (51.67)	15,366 (52.47)	482 (33.00)	
Marital status, n (%)				0.620
Married or living with a partner	19,271 (66.22)	18,278 (66.25)	993 (65.49)	
Living alone	11,913 (33.78)	11,302 (33.75)	611 (34.51)	
Race/ethnicity, n (%)				< 0.001
Non-Hispanic white	4,150 (6.25)	4,009 (6.39)	141 (2.87)	
Non-Hispanic black	2,452 (4.76)	2,363 (4.86)	89 (2.60)	
Mexican American	15,205 (72.62)	14,125 (72.16)	1,080 (83.41)	
Other	9,377 (16.37)	9,083 (16.59)	294 (11.11)	
Education level, n (%)				< 0.001
Below high school	6,696 (13.44)	6,211 (13.02)	485 (23.11)	
High school	7,089 (22.91)	6,701 (22.76)	388 (26.36)	
Above high school	17,399 (63.65)	16,668 (64.22)	731 (50.53)	
PIR, n (%)				0.813
≥ 1	25,787 (88.52)	24,456 (88.51)	1,331 (88.73)	
< 1	5,397 (11.48)	5,124 (11.49)	273 (11.27)	
BMI (kg/m^2^), n (%)				< 0.001
Normal weight	9,244 (30.81)	8,836 (31.14)	408 (23.23)	
Low weight	405 (1.37)	389 (1.39)	16 (0.79)	
Overweight	10,849 (34.65)	10,259 (34.60)	590 (35.75)	
Obesity	10,686 (33.17)	10,096 (32.87)	590 (40.23)	
Smoking, n (%)				< 0.001
No	16,828 (54.48)	16,264 (55.35)	564 (34.41)	
Yes	14,356 (45.52)	13,316 (44.65)	1,040 (65.59)	
Stroke, n (%)				< 0.001
No	29,853 (96.95)	28,502 (97.44)	1,351 (85.53)	
Yes	1,331 (3.05)	1,078 (2.56)	253 (14.47)	
Hypertension, n (%)				< 0.001
No	18,804 (65.46)	18,400 (67.11)	404 (27.46)	
Yes	12,380 (34.54)	11,180 (32.89)	1,200 (72.54)	
Hyperlipidemia, n (%)				< 0.001
No	18,942 (62.51)	18,497 (64.09)	445 (25.84)	
Yes	12,242 (37.49)	11,083 (35.91)	1,159 (74.16)	
Diabetes, n (%)				< 0.001
No	26,874 (89.95)	25,817 (90.90)	1,057 (68.02)	
Yes	4,310 (10.05)	3,763 (9.10)	547 (31.98)	
Supplements taken, n (%)				< 0.001
No	17,047 (57.49)	16,028 (57.14)	1,019 (65.60)	
Yes	14,137 (42.51)	13,552 (42.86)	585 (34.40)	
Alcohol (g/d), Median (IQR)	0.00 (0.00, 9.35)	0.00 (0.00, 9.35)	0.00 (0.00, 3.25)	< 0.001
HDL-C (mmol/L), Median (IQR)	1.32 (1.09, 1.60)	1.32 (1.09, 1.63)	1.16 (0.98, 1.45)	< 0.001
TC (mmol/L), Median (IQR)	5.02 (4.34, 5.74)	5.04 (4.37, 5.74)	4.40 (3.75, 5.20)	< 0.001
ALA (g/d), Median (IQR)	1.36 (0.91, 2.00)	1.37 (0.92, 2.01)	1.27 (0.85, 1.89)	< 0.001
DPA (g/d), Median (IQR)	0.01 (0.01, 0.03)	0.01 (0.01, 0.03)	0.01 (0.00, 0.02)	0.017
ETA (g/d), Median (IQR)	0.12 (0.07, 0.19)	0.12 (0.07, 0.19)	0.11 (0.06, 0.18)	0.001
EPA (g/d), Median (IQR)	0.01 (0.00, 0.02)	0.01 (0.00, 0.02)	0.01 (0.00, 0.02)	0.990
DHA (g/d), Median (IQR)	0.03 (0.01, 0.06)	0.03 (0.01, 0.06)	0.02 (0.01, 0.06)	0.633
Omega-3 (g/d), Median (IQR)	1.62 (1.10, 2.35)	1.62 (1.10, 2.36)	1.52 (1.03, 2.26)	< 0.001

Abbreviations: CHD, coronary heart disease; IQR, interquartile range; PIR, poverty income ratio; BMI, body mass index; HDL-C, high-density lipoprotein cholesterol; TC, total cholesterol; ALA, α-linolenic acid; DPA, docosapentaenoic acid; ETA, eicosatetraenoic acid; EPA, eicosapentaenoic acid; DHA, docosahexenoic acid.

* n is unweighted, percentages and median (IQR) are weighted, and *P*-values are calculated from the weighted data.

### Relationship between dietary omega-3 intake and CHD

**Fig 2:**
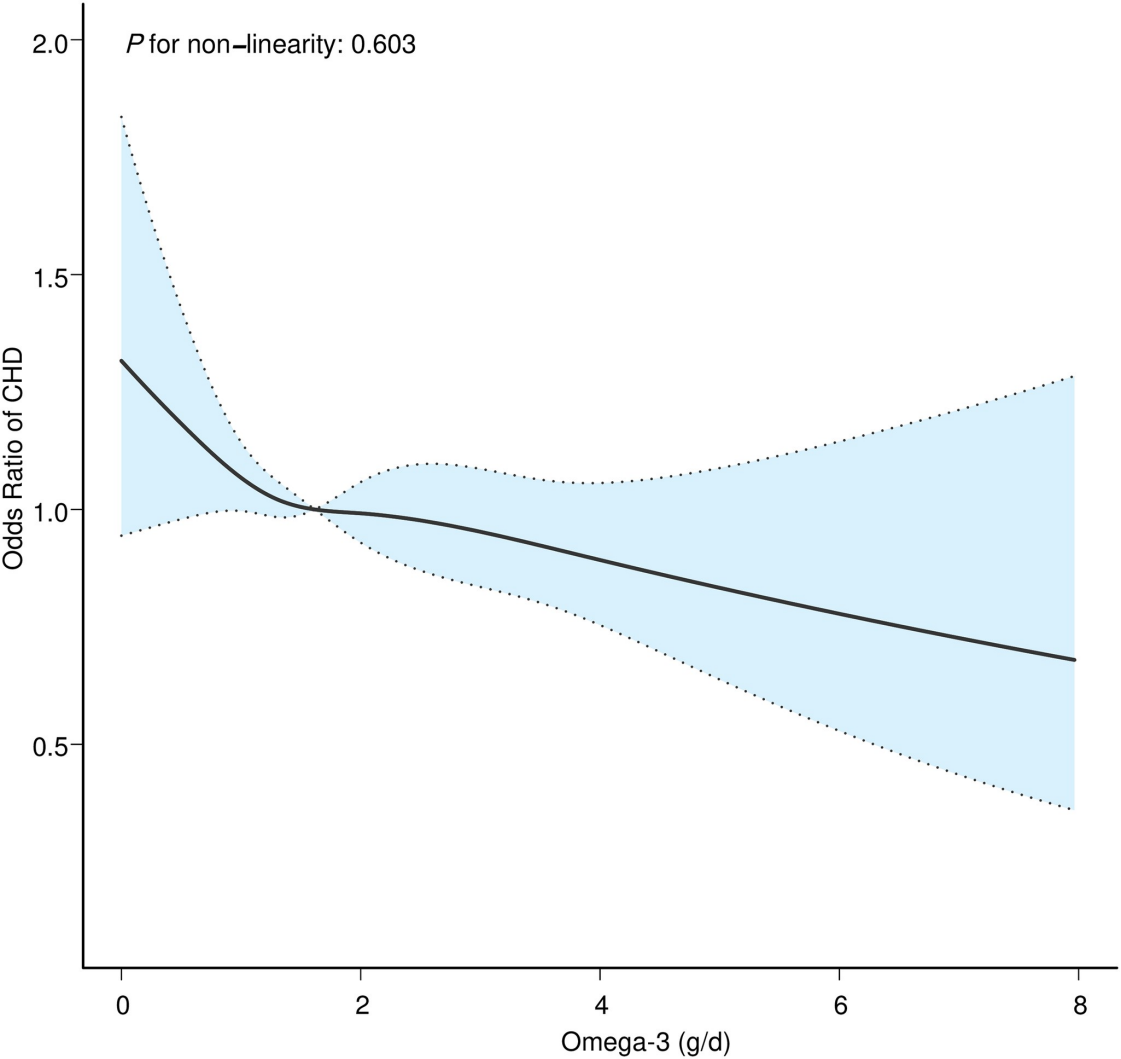
Dose-response relationship between dietary omega-3 intake and CHD. The solid line indicates the estimated risk of CHD, and the dashed line indicates the fitted 95% CI.

After stratifying omega-3 intake by quintile and performing multifactorial logistic regression, total omega-3 intake was negatively associated with CHD when not adjusted for confounders (Trend p = 0.006) (Table 2). The effect of dietary omega-3 intake on CHD remained significant after adjustment for potential confounders (Trend p = 0.032). When unadjusted, the risk of developing CHD decreased by 26% intake level in Q5 compared to Q1 (0.74 (0.60, 0.91)). After adjusting models 1, 2, and 3, respectively, the risk of CHD at intake up to Q5 remained significantly lower than at Q1 (0.76 (0.61, 0.95), 0.79 (0.63, 1.00), 0.76 (0.60, 0.96)). Therefore, we used RCS analysis further to observe the relationship between dietary omega-3 intake and CHD. The RCS results showed (Fig 2) that dietary omega-3 intake negatively linearly correlated with CHD (*P* for nonlinear = 0.603) after adjusting for all confounders (Model 3). This is consistent with our logistic regression results and increases the reliability of the results.

**Table 2:** Association between dietary omega-3 intake and CHD in different models.

Variable	N	Crude OR (95%CI)	P-value	Model 1 OR (95%CI)	P-value	Model 2 OR (95%CI)	P-value	Model 3 OR (95%CI)	P-value
Omega-3 (g/d)									
Q1 (≤ 1.00)	6,285	1(Ref)		1(Ref)		1(Ref)		1(Ref)	
Q2 (1.01–1.41)	6,223	0.90 (0.75, 1.09)	0.293	0.91 (0.74, 1.11)	0.345	0.91 (0.74, 1.14)	0.424	0.90 (0.72, 1.12)	0.356
Q3 (1.42–1.87)	6,266	0.86 (0.70, 1.07)	0.171	0.92 (0.74, 1.14)	0.442	0.93 (0.75, 1.17)	0.552	0.91 (0.73, 1.14)	0.404
Q4 (1.88–2.57)	6,195	0.75 (0.60, 0.92)	0.007	0.78 (0.62, 0.97)	0.028	0.78 (0.62, 0.99)	0.044	0.80 (0.63, 1.01)	0.065
Q5 (≥ 2.58)	6,215	0.74 (0.60, 0.91)	0.005	0.76 (0.61, 0.95)	0.018	0.79 (0.63, 1.00)	0.049	0.76 (0.60, 0.96)	0.022
Trend p		0.006		0.022		0.054		0.032	

Abbreviations: Q1 to Q5, quintile 1 to 5; OR, odds ratio; CI, confidence interval; Ref, reference.

Crude: unadjusted.

Model 1: adjusted for age + sex + race/ethnicity + education + marital status + PIR.

Model 2: adjusted for model 1 + smoking + alcohol intake + stroke + hypertension + hyperlipidemia + diabetes.

Model 3: adjusted for model 2 + dietary supplements + BMI + HDL-C + TC.

In addition, to explore the association of single omega-3 components with CHD, we performed a logistic regression analysis for each omega-3 component (S2 Table). When unadjusted, ALA, DPA, and ETA all reduced the risk of CHD at Q5 compared with Q1 (0.71 (0.57, 0.89), 0.64 (0.50, 0.81), 0.69 (0.55, 0.87)). When analyzing the adjusted quintiles, we still observed significant decreasing trends in ALA, DPA, and ETA (0.73 (0.57, 0.94), 0.70 (0.54, 0.92), 0.66 (0.50, 0.85)). Although there was a slight reduction effect of EPA and DHA on CHD risk before and after adjustment, the confidence interval (CI) was wide, and the effect was insignificant (Trend p > 0.05). RCS analysis revealed that all omega-3 components have a negative linear correlation with CHD (*P* for nonlinear > 0.05) (S1 Fig).

### Subgroup analysis of the association between omega-3 intake and CHD

**Fig 3:**
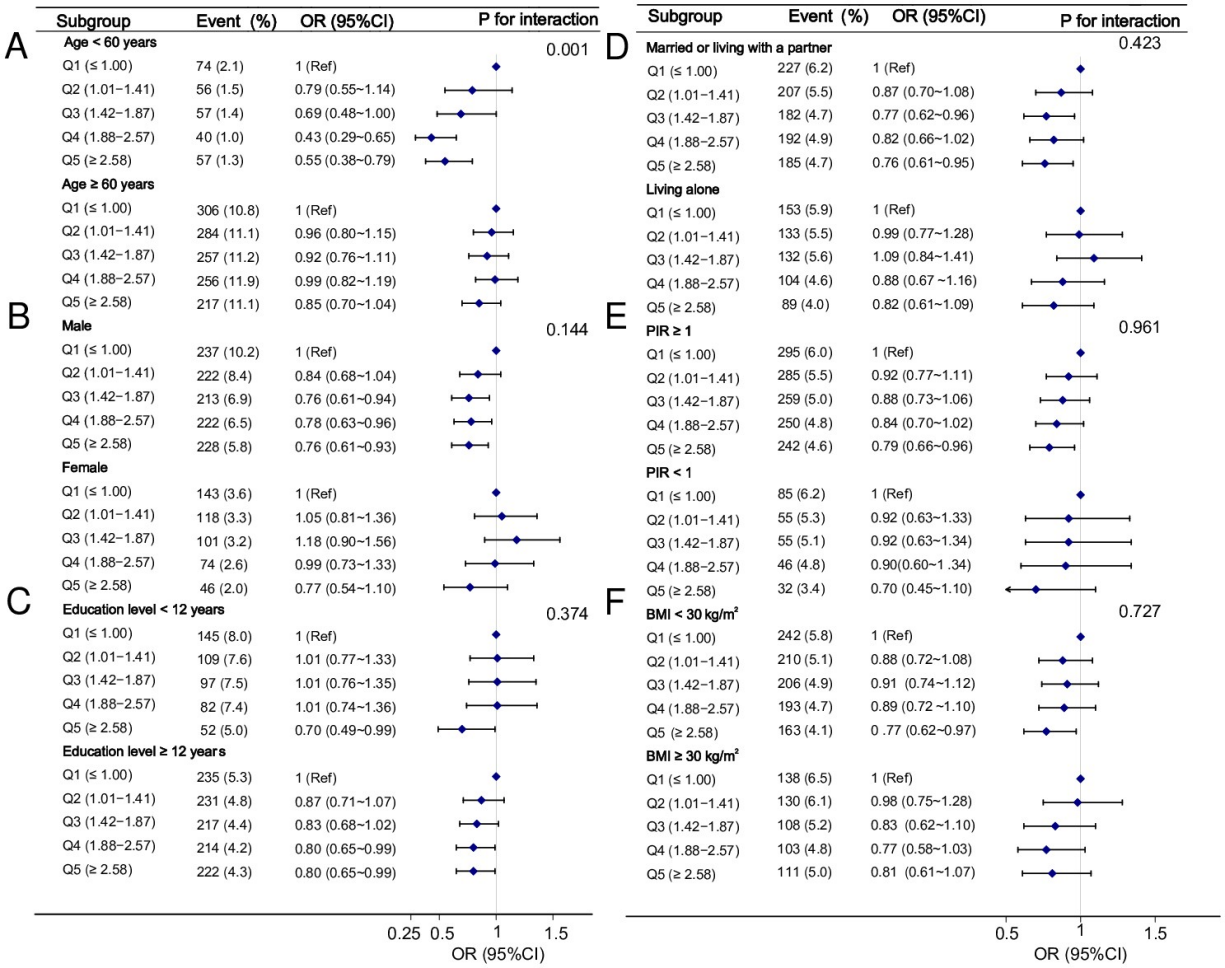
Association between dietary omega-3 intake and CHD in different subgroups. Abbreviations: Q1 to Q5, quintile 1 to 5; OR, odds ratio; CI, confidence interval; Ref, reference; PIR, poverty income ratio; BMI, body mass index.

A subgroup analysis was performed according to age, sex, education level, marital status, PIR, and BMI to explore the stability of the relationship between dietary omega-3 intake and CHD. We adjusted for variables other than stratification variables in Model 3. Subgroup analysis revealed an interaction between omega-3 and CHD in the age group (P for interaction = 0.001). The effect of dietary omega-3 intake in reducing CHD risk was more significant in those aged < 60 years than those aged ≥ 60 years (0.55 (0.38, 0.79) vs 0.85 (0.70,1.04)) (Fig 3). There was no interaction between other subgroup variables on the association between omega-3 and CHD (P for interaction > 0.05) (Fig 3).

### Sensitivity analysis

In addition, we conducted a sensitivity analysis to ensure the stability and reliability of the results. We performed regression analysis after excluding extreme values of energy intake that were beyond typical ranges (< 500 kcal/d or > 4,000 kcal/d for females; < 800 kcal/d or > 6,000 kcal/d for males) or extreme BMI (< 18.5 kg/m^2^ or > 30 kg/m^2^), respectively. The results did not change substantially (S3–S6 Tables).

## Discussion

The total dietary intake of omega-3, ALA, DPA, and ETA was negatively linked to the risk of CHD among the U.S. adults in our study. After adjusting for all confounders, multivariate logistic regression results showed that higher dietary omega-3 intake was still independently associated with a lower risk of CHD. Meanwhile, the results of RCS analysis further confirmed the negative association of dietary omega-3 intake with CHD. Sensitivity analysis verified the stability of the association. In contrast, the strength of the association of DHA or EPA with CHD was not significant before and after adjustment for confounding factors. According to the age-based stratified analysis, individuals under the age of 60 were more likely to benefit from omega-3 intake.

The relationship between omega-3 and cardiovascular events has long been extensively studied, but there is wide variation in the findings. In an open-label (JELIS) study of 9,326 hypercholesterolemic patients in the EPA group after a 4.6-year intervention with 1,800 mg EPA daily combined with a statin, patients experienced a 19% reduction in coronary events [35]. Another randomized controlled trial (RCT) study reached similar conclusions. In the REDUCE-IT study, Bhatt DL et al. reported a 25% reduction in cardiovascular events after 4 g of icosapent ethyl (highly purified EPA) daily intake for 4.9 years [36]. According to a multi-ethnic cohort study of 2,837 U.S. adults, both dietary and circulating EPA or DHA were negatively associated with CVD, but the same results were not observed in ALA [37]. A Danish cohort study similarly confirmed that dietary EPA, DHA, or EPA + DHA intake was negatively associated with total atherosclerotic cardiovascular disease (ASCVD) incidence, but no correlation was found in ALA [38].

However, recent RCTs have no evidence that EPA + DHA supplements can prevent cardiovascular events [39, 40]. According to the VITAL study, which included 25,871 subjects without prior CVD risk factors, there was no difference between the trial and placebo groups regarding cardiovascular events after 5.3 years of subjects taking 840 mg EPA + DHA and 2,000 IU vitamin D per day [39]. Another RCT revealed similar findings. In the STRENGTH study conducted by Nicholls SJ et al., 13,078 adult participants at high risk of cardiovascular events were randomly assigned to 4 g/d omega-3 (EPA + DHA) or corn oil, which showed a non-statistically significant difference in the incidence of adverse cardiovascular events of 12% and 12.2% in the two groups, respectively. The trial was terminated early because of low clinical benefit and increased atrial fibrillation risk [40].

Differences in study results may be attributed to differences in the characteristics of the subjects (e.g., age, race, etc.), baseline omega-3 status, dosage and measurement methods, treatment adherence, and combination medications. The negative correlation between omega-3 and CHD found in this study was more robust in individuals under the age of 60. This may be related not only to the higher demand for and intake of fatty acids in young and middle-aged adults but also to their faster metabolic rate and more active conversion of fatty acids in the bodies in young and middle-aged adults. The effect of age has also been confirmed in some studies. A cohort study of middle-aged Japanese adults showed that cardiovascular arrest death and fatal coronary events, but not overall cardiovascular disease risk, were associated with plasma omega-3 levels (EPA + DHA + DPA) [41]. Therefore, in approaching these results, we need to consider not only the inevitable individual differences between populations but also a more detailed stratification of the disease may be necessary. Due to the lack of information on CHD sub-diagnosis in the database, the disease typing of CHD was not studied in this study.

Furthermore, these studies assessed only the effects of EPA, DHA, or ALA. They did not examine other components of omega-3, which may lead to an underestimation of the association between omega-3 and CHD. This study integrated ALA, ETA, DPA, EPA, and DHA to obtain the total dietary omega-3 intake. In addition, we also performed association analysis for the single components of omega-3 separately. In the multivariate logistic regression of total omega-3 and its single components, we found a significant negative linear relationship between total omega-3, ALA, ETA, and DPA intakes with CHD. No significant correlation with CHD was found when the EPA and DHA were analyzed separately. This is consistent with the findings of Manson JE et al. and Nicholls SJ et al [39, 40]. A recent retrospective study also showed that flaxseed oil (400 mg ALA/1,000 mg) was more effective at reducing blood hs-CRP levels than fish oil (250 mg EPA and 150 mg DHA/1,000 mg) in patients with CHD [42]. This inconsistency in results may be related to two things. First, the intake of EPA and DHA in the included population is generally small. EPA and DHA account for a relatively small proportion of the total omega-3 intake, and their effects may be masked. Second, because other fatty acids, such as ALA, can be converted into DHA and EPA in the body [43, 44], the intake analysis does not accurately reflect the amount of fatty acids in the blood.

ALA is the precursor substance of all omega-3 polyunsaturated fatty acids and can be synthesized in the body as EPA, which is then converted to DHA by β-oxidation, thus exerting anti-inflammatory, inflammation-resolving, anti-thrombotic, and triglyceride-lowering effects similar to DHA and EPA [45–47]. DHA and EPA have been proven to have anti-inflammatory benefits [48, 49] that improve blood circulation and protect vascular function by reducing inflammation-causing factors such as ICAM-1, VCAM-1, and E-selectin through the inhibition of endothelial cell activation [50, 51]. DHA and EPA also exhibit anti-inflammatory effects through derived lysins and protectins [52, 53]. These lysins and protectins reduce the production of pro-inflammatory markers and slow the progression of atherosclerosis [54]. In addition, DHA and EPA reduce the production of TXA2 and PGI2 by reducing the synthesis of inflammatory arachidonic acid. This promotes vasodilation, inhibits platelet aggregation, reduces thrombosis, and exerts a cardioprotective effect [55–57]. Like DHA and EPA, DPA belongs to the long-chain polyunsaturated fatty acid family, mainly derived from EPA. It can be interconverted with EPA, with effects similar to EPA [58]. As a precursor to EPA, ETA may respond more accurately to omega-3 polyunsaturated fatty acid status than EPA [59]. The anti-inflammatory effects of ETA are attributed to its ability to inhibit the oxidation of arachidonic acid to prostaglandins through the lipoxygenase and cyclooxygenase (COX) pathways [60]. There are few studies on the association of ETA with CHD. A study exploring the association between ETA and heart failure (HF) showed that plasma ETA concentrations were negatively correlated with hs-CRP and NT-proBNT levels in HF patients and that low levels of ETA were strongly linked to increased severity of disease and mortality in HF patients [59]. In conclusion, the effect of omega-3 intake on CHD remains inconclusive, and current studies on omega-3’s mechanism of action on cardiovascular effects cannot fully explain the results of clinical studies. Therefore, more basic studies and extensive cohort studies are needed to explore this topic further.

### Limitations

A few limitations apply to this study. First, this study is cross-sectional, which can only confirm the correlation between omega-3 intake and CHD risk but cannot infer causality yet. Second, the diagnosis of CHD was based on the study subjects’ self-reporting, and the lack of objective diagnostic criteria may lead to reporting bias. Third, dietary fatty acid intake was determined by recalling the diet for 24 hours. Despite using an average of two 24-hour recalls, we could not avoid possible recall bias and measurement error. In addition, we could not track dietary fatty acid intake over time due to database limitations. Fourth, although there is no clear evidence of interactions among the omega-3 components, the pooled analysis of the five omega-3 components in this study does not exclude the possibility of interactions among the components, which could affect the assessment of CHD risk. Fifth, the participants in this study were U.S. adults, and more research is needed to confirm whether the results can be extrapolated to other ethnic groups. Sixth, despite adjusting for as many influences as possible, confounding effects of unincluded or unknown factors could not be excluded. The association between omega-3 intake and CHD is complex. Our findings may provide ideas and evidence to support future studies of the association between the two.

## Conclusion

It was found that the total dietary omega-3 intake and CHD risk were negatively correlated, with a relationship more apparent in individuals under the age of 60. Therefore, more extensive and higher-quality studies based on population characteristics and omega-3 types remain necessary. By integrating evidence from more detailed studies and identifying commonalities and differences between different studies, we can summarize the population and dietary intake characteristics to provide more rationalized dietary recommendations for different populations.

## Supporting information

S1 Table Population characteristics by the quintile of dietary omega-3 intake. (DOCX)

S2 Table Association of different components of omega-3 with CHD. (DOCX)

S3 Table Association between dietary omega-3 intake and CHD after exclusion of energy extremes. (DOCX)

S4 Table Association between dietary omega-3 intake of each component and CHD after exclusion of energy extremes. (DOCX)

S5 Table Association between dietary omega-3 intake and CHD after exclusion of BMI extremes. (DOCX)

S6 Table Association between dietary omega-3 component intake and CHD after exclusion of BMI extremes. (DOCX)

S1 Fig Dose-response relationship between dietary ALA(A), DHA(B), ETA (C), EPA(D), DHA(E) intake and CHD. (DOCX)
