# Supplementary material for: Association between dietary omega-3 intake and coronary heart disease among American adults: The NHANES, 1999–2018
Source: PLoS One. 2023 Dec 20;18(12):e0294861. doi: 10.1371/journal.pone.0294861 (PMC10732455; doi:10.1371/journal.pone.0294861)
Supplement: S1 Table — (DOCX) [file pone.0294861.s001.docx]

**Table S1.** **Population characteristics by the quintile of dietary omega-3 intake.**

|  |  | **Omega-3 intake, g/d** | | | | | | |
| --- | --- | --- | --- | --- | --- | --- | --- | --- |
| **Variables** | | **Total** | **Q1**  **(**≤ **1.00)** | **Q2**  **(1.01-1.41)** | **Q3**  **(1.42-1.87)** | **Q4**  **(1.88-2.57)** | **Q5**  **(**≥ **2.58)** | **P-value** |
| N | | 31,184 | 6,285 | 6,223 | 6,266 | 6,195 | 6,215 |  |
| Age (years), Median (IQR) | | 49.00 (36.00, 61.00) | 50.00 (38.00, 64.00) | 50.00 (37.00, 63.00) | 48.00 (36.00, 61.00) | 48.00 (36.00, 60.00) | 48.00 (36.00, 59.00) | <0.001 |
| Age (years), n (%) | | |  |  |  |  |  | <0.001 |
|  | 20-44 years | 11,315 (40.62) | 1,958 (37.50) | 2,090 (37.88) | 2,351 (42.74) | 2,393 (41.99) | 2,523 (42.44) |  |
|  | 45-64 years | 11,352 (39.39) | 2,181 (37.89) | 2,222 (39.64) | 2,287 (38.35) | 2,291 (39.65) | 2,371 (41.17) |  |
|  | ≥ 65 years | 8,517 (19.98) | 2,146 (24.61) | 1,911 (22.49) | 1,628 (18.91) | 1,511 (18.35) | 1,321 (16.39) |  |
| Sex, n (%) | |  |  |  |  |  |  | <0.001 |
|  | Male | 15,336 (48.33) | 2,320 (34.00) | 2,643 (39.93) | 3,074 (47.77) | 3,400 (53.88) | 3,899 (63.22) |  |
|  | Female | 15,848 (51.67) | 3,965 (66.00) | 3,580 (60.07) | 3,192 (52.23) | 2,795 (46.12) | 2,316 (36.78) |  |
| Marital status, n (%) | | |  |  |  |  |  | <0.001 |
|  | Married or living with a partner | 19,271 (66.22) | 3,689 (63.55) | 3,788 (65.82) | 3,888 (65.63) | 3,943 (67.38) | 3,963 (68.28) |  |
|  | Living alone | 11,913 (33.78) | 2,596 (36.45) | 2,435 (34.18) | 2,378 (34.37) | 2,252 (32.62) | 2,252 (31.72) |  |
| Race/ethnicity, n (%) | | |  |  |  |  |  | 0.553 |
|  | Non, Hispanic white | 4,150 (6.25) | 922 (5.93) | 833 (6.19) | 839 (6.35) | 793 (6.38) | 763 (6.34) |  |
|  | Non, Hispanic black | 2,452 (4.76) | 509 (5.19) | 518 (4.88) | 485 (4.45) | 496 (4.87) | 444 (4.49) |  |
|  | Mexican American | 15,205 (72.62) | 3,044 (71.90) | 3,070 (72.76) | 3,043 (72.30) | 2,992 (72.84) | 3,056 (73.21) |  |
|  | Other | 9,377 (16.37) | 1,810 (16.98) | 1,802 (16.16) | 1,899 (16.89) | 1,914 (15.91) | 1,952 (15.96) |  |
| Education level, n (%) | | |  |  |  |  |  | <0.001 |
|  | Below high school | 6,696 (13.44) | 1,815 (17.90) | 1,436 (14.87) | 1,296 (12.99) | 1,110 (11.19) | 1,039 (10.95) |  |
|  | High school | 7,089 (22.91) | 1,513 (25.29) | 1,446 (23.60) | 1,403 (22.43) | 1,374 (22.39) | 1,353 (21.21) |  |
|  | Above high school | 17,399 (63.65) | 2,957 (56.81) | 3,341 (61.53) | 3,567 (64.58) | 3,711 (66.42) | 3,823 (67.84) |  |
| PIR, n (%) | |  |  |  |  |  |  | <0.001 |
|  | ≥ 1 | 25,787 (88.52) | 4,914 (84.77) | 5,179 (89.12) | 5,179 (88.09) | 5,244 (89.96) | 5,271 (90.16) |  |
|  | < 1 | 5,397 (11.48) | 1,371 (15.23) | 1,044 (10.88) | 1,087 (11.91) | 951 (10.04) | 944 (9.84) |  |
| BMI (kg/m^2^), n (%) | | |  |  |  |  |  | <0.001 |
|  | Normal weight | 9,244 (30.81) | 1,944 (33.96) | 1,873 (31.36) | 1,850 (30.70) | 1,817 (30.14) | 1,760 (28.38) |  |
|  | Low weight | 405 (1.37) | 81 (1.24) | 84 (1.50) | 83 (1.37) | 73 (1.22) | 84 (1.49) |  |
|  | Overweight | 10,849 (34.65) | 2,128 (32.74) | 2,133 (33.70) | 2,248 (35.85) | 2,179 (35.66) | 2,161 (35.02) |  |
|  | Obesity | 10,686 (33.17) | 2,132 (32.05) | 2,133 (33.43) | 2,085 (32.07) | 2,126 (32.97) | 2,210 (35.12) |  |
| Smoking, n (%) | |  |  |  |  |  |  | 0.138 |
|  | No | 16,828 (54.48) | 3,341 (52.89) | 3,444 (55.35) | 3,404 (55.86) | 3,340 (54.12) | 3,299 (54.10) |  |
|  | Yes | 14,356 (45.52) | 2,944 (47.11) | 2,779 (44.65) | 2,862 (44.14) | 2,855 (45.88) | 2,916 (45.90) |  |
| Stroke, n (%) | |  |  |  |  |  |  | <0.001 |
|  | No | 29,853 (96.95) | 5,906 (95.59) | 5,905 (96.14) | 6,028 (97.32) | 5,982 (97.58) | 6,032 (97.87) |  |
|  | Yes | 1,331 (3.05) | 379 (4.41) | 318 (3.86) | 238 (2.68) | 213 (2.42) | 183 (2.13) |  |
| Hypertension, n (%) | | |  |  |  |  |  | <0.001 |
|  | No | 18,804 (65.46) | 3,514 (61.89) | 3,680 (64.68) | 3,835 (66.26) | 3,880 (67.17) | 3,895 (66.81) |  |
|  | Yes | 12,380 (34.54) | 2,771 (38.11) | 2,543 (35.32) | 2,431 (33.74) | 2,315 (32.83) | 2,320 (33.19) |  |
| Hyperlipidemia, n (%) | | |  |  |  |  |  | 0.362 |
|  | No | 18,942 (62.51) | 3,699 (61.11) | 3,776 (63.07) | 3,817 (62.84) | 3,792 (63.26) | 3,858 (62.14) |  |
|  | Yes | 12,242 (37.49) | 2,586 (38.89) | 2,447 (36.93) | 2,449 (37.16) | 2,403 (36.74) | 2,357 (37.86) |  |
| Diabetes, n (%) | |  |  |  |  |  |  | 0.010 |
|  | No | 26,874 (89.95) | 5,240 (88.39) | 5,380 (90.12) | 5,440 (90.39) | 5,363 (90.01) | 5,451 (90.64) |  |
|  | Yes | 4,310 (10.05) | 1,045 (11.61) | 843 (9.88) | 826 (9.61) | 832 (9.99) | 764 (9.36) |  |
| Supplements taken, n (%) | | |  |  |  |  |  | 0.247 |
|  | No | 17,047 (57.49) | 3,326 (56.67) | 3,391 (57.23) | 3,466 (58.29) | 3,404 (56.53) | 3,460 (58.59) |  |
|  | Yes | 14,137 (42.51) | 2,959 (43.33) | 2,832 (42.77) | 2,800 (41.71) | 2,791 (43.47) | 2,755 (41.41) |  |
| Alcohol (g/d), Median (IQR) | | 0.00 (0.00, 9.35) | 0.00 (0.00, 0.80) | 0.00 (0.00, 7.00) | 0.00 (0.00, 9.60) | 0.00 (0.00, 11.15) | 0.00 (0.00, 14.00) | <0.001 |
| HDL-C (mmol/L), Median (IQR) | | 1.32 (1.09, 1.60) | 1.34 (1.09, 1.63) | 1.34 (1.09, 1.63) | 1.34 (1.09, 1.63) | 1.29 (1.09, 1.60) | 1.29 (1.06, 1.58) | <0.001 |
| TC (mmol/L), Median (IQR) | | 5.02 (4.34, 5.74) | 5.04 (4.37, 5.79) | 5.04 (4.34, 5.77) | 4.99 (4.32, 5.72) | 5.04 (4.37, 5.72) | 4.97 (4.29, 5.69) | <0.001 |
| ALA (g/d), Median (IQR) | | 1.36 (0.91, 2.00) | 0.64 (0.49, 0.76) | 1.04 (0.93, 1.15) | 1.41 (1.29, 1.54) | 1.90 (1.72, 2.09) | 2.84 (2.45, 3.47) | <0.001 |
| DPA (g/d), Median (IQR) | | 0.01 (0.01, 0.03) | 0.01 (0.00, 0.01) | 0.01 (0.00, 0.02) | 0.01 (0.01, 0.02) | 0.02 (0.01, 0.03) | 0.03 (0.01, 0.05) | <0.001 |
| ETA (g/d), Median (IQR) | | 0.12 (0.07, 0.19) | 0.06 (0.04, 0.10) | 0.10 (0.06, 0.15) | 0.12 (0.08, 0.18) | 0.15 (0.09, 0.21) | 0.19 (0.13, 0.29) | <0.001 |
| EPA (g/d), Median (IQR) | | 0.01 (0.00, 0.02) | 0.00 (0.00, 0.01) | 0.01 (0.00, 0.01) | 0.01 (0.00, 0.02) | 0.01 (0.00, 0.03) | 0.02 (0.01, 0.08) | <0.001 |
| DHA (g/d), Median (IQR) | | 0.03 (0.01, 0.06) | 0.01 (0.00, 0.02) | 0.02 (0.00, 0.04) | 0.03 (0.01, 0.06) | 0.04 (0.01, 0.08) | 0.06 (0.02, 0.17) | <0.001 |

Abbreviations: CHD, coronary heart disease; Q1 to Q5, quintile 1 to 5; IQR, interquartile range; PIR, poverty income ratio; BMI, body mass index; HDL-C, high-density lipoprotein cholesterol; TC, total cholesterol; ALA, α-linolenic acid; DPA, docosapentaenoic acid; ETA, eicosatetraenoic acid; EPA, eicosapentaenoic acid; DHA, docosahexenoic acid.
