# Supplementary material for: Association between dietary omega-3 intake and coronary heart disease among American adults: The NHANES, 1999–2018
Source: PLoS One. 2023 Dec 20;18(12):e0294861. doi: 10.1371/journal.pone.0294861 (PMC10732455; doi:10.1371/journal.pone.0294861)
Supplement: S2 Table — (DOCX) [file pone.0294861.s002.docx]

**Table S2. Association of different components of omega-3 with CHD.**

| **Variables** | | **N** | **Crude**  **OR (95%CI)** | **P-value** | **Model 1**  **OR (95%CI)** | **P-value** | **Model 2**  **OR (95%CI)** | **P-value** | **Model 3**  **OR (95%CI)** | | **P-value** |
| --- | --- | --- | --- | --- | --- | --- | --- | --- | --- | --- | --- |
| ALA (g/d) | |  | | | | | | |  |  |  |
| Q1 (≤ 0.82) | | 5,986 | 1(Ref) |  | 1(Ref) |  | 1(Ref) |  | 1(Ref) | |  |
| Q2 (0.83-1.18) | | 6,569 | 0.83 (0.69, 1.00) | 0.052 | 0.84 (0.69, 1.03) | 0.095 | 0.83 (0.67, 1.02) | 0.078 | 0.81 (0.66, 1.00) | | 0.053 |
| Q3 (1.19-1.58) | | 6,430 | 0.90 (0.75, 1.10) | 0.308 | 0.95 (0.77, 1.16) | 0.602 | 0.98 (0.78, 1.22) | 0.832 | 0.96 (0.77, 1.19) | | 0.690 |
| Q4 (1.59-2.19) | | 6,383 | 0.72 (0.58, 0.88) | 0.002 | 0.77 (0.62, 0.96) | 0.020 | 0.77 (0.61, 0.97) | 0.026 | 0.76 (0.60, 0.97) | | 0.027 |
| Q5 (≥ 2.20) | | 5,816 | 0.71 (0.57, 0.89) | 0.003 | 0.74 (0.58, 0.95) | 0.017 | 0.77 (0.60, 0.98) | 0.038 | 0.73 (0.57, 0.94) | | 0.016 |
| Trend p | |  | 0.004 |  | 0.021 |  | 0.048 |  | 0.024 | |  |
| DPA (g/d) | |  | | | | | | | | |  |
| Q1 (≤ 0.004) | | 6,020 | 1(Ref) |  | 1(Ref) |  | 1(Ref) |  | 1(Ref) | |  |
| Q2 (0.005-0.011) | | 6,527 | 0.79 (0.64, 0.96) | 0.019 | 0.91 (0.74, 1.11) | 0.351 | 0.86 (0.70, 1.06) | 0.151 | 0.80 (0.64, 1.00) | | 0.052 |
| Q3 (0.012-0.018) | | 6,311 | 0.78 (0.63, 0.96) | 0.021 | 0.97 (0.78, 1.19) | 0.750 | 0.95 (0.77, 1.17) | 0.617 | 0.88 (0.71, 1.09) | | 0.254 |
| Q4 (0.019-0.031) | | 6,324 | 0.69 (0.56, 0.86) | 0.001 | 0.81 (0.65, 1.02) | 0.072 | 0.76 (0.61, 0.96) | 0.021 | 0.70 (0.56, 0.89) | | 0.003 |
| Q5 (≥ 0.032) | | 6,002 | 0.64 (0.50, 0.81) | <0.001 | 0.78 (0.61, 1.01) | 0.057 | 0.76 (0.58, 0.99) | 0.043 | 0.70 (0.54, 0.92) | | 0.012 |
| Trend p | |  | <0.001 |  | 0.042 |  | 0.038 |  | 0.013 | |  |
| ETA (g/d) | |  | | | | | | | | |  |
| Q1 (≤ 0.060) | | 5,945 | 1(Ref) |  | 1(Ref) |  | 1(Ref) |  | 1(Ref) | |  |
| Q2 (0.061-0.101) | | 6,512 | 0.86 (0.71, 1.05) | 0.136 | 0.90 (0.73, 1.12) | 0.351 | 0.89 (0.72, 1.10) | 0.284 | 0.88 (0.71, 1.10) | | 0.261 |
| Q3 (0.102-0.147) | | 6,429 | 0.88 (0.73, 1.05) | 0.158 | 0.97 (0.79, 1.18) | 0.754 | 0.92 (0.75, 1.13) | 0.444 | 0.92 (0.75, 1.12) | | 0.410 |
| Q4 (0.148-0.217) | | 6,462 | 0.81 (0.66, 0.99) | 0.039 | 0.83 (0.67, 1.05) | 0.118 | 0.81 (0.63, 1.03) | 0.086 | 0.80 (0.62, 1.02) | | 0.074 |
| Q5 (≥ 0.218) | | 5,836 | 0.69 (0.55, 0.87) | 0.002 | 0.73 (0.56, 0.94) | 0.015 | 0.67 (0.51, 0.87) | 0.003 | 0.66 (0.50, 0.85) | | 0.002 |
| Trend p | |  | 0.001 |  | 0.008 |  | 0.002 |  | 0.001 | |  |
| EPA (g/d) | |  | | | | | | | | |  |
| Q1 (≤ 0.003) | | 6,598 | 1(Ref) |  | 1(Ref) |  | 1(Ref) |  | 1(Ref) | |  |
| Q2 (0.004-0.006) | | 6,439 | 0.84 (0.70, 1.02) | 0.085 | 0.85 (0.70, 1.03) | 0.096 | 0.80 (0.66, 0.98) | 0.035 | 0.79 (0.65, 0.96) | | 0.021 |
| Q3 (0.007-0.011) | | 5,991 | 0.71 (0.57, 0.90) | 0.004 | 0.78 (0.61, 0.98) | 0.038 | 0.77 (0.60, 0.98) | 0.034 | 0.73 (0.57, 0.93) | | 0.011 |
| Q4 (0.012-0.029) | | 5,940 | 0.88 (0.68, 1.13) | 0.317 | 0.98 (0.75, 1.29) | 0.898 | 0.96 (0.73, 1.27) | 0.773 | 0.91 (0.69, 1.21) | | 0.530 |
| Q5 (≥ 0.030) | | 6,216 | 0.84 (0.69, 1.02) | 0.073 | 0.84 (0.69, 1.02) | 0.087 | 0.86 (0.71, 1.05) | 0.141 | 0.84 (0.69, 1.02) | | 0.075 |
| Trend p | |  | 0.614 |  | 0.427 |  | 0.782 |  | 0.695 | |  |
| DHA (g/d) | |  | | | | | | | | |  |
| Q1 (≤ 0.005) | | 6,096 | 1(Ref) |  | 1(Ref) |  | 1(Ref) |  | 1(Ref) | |  |
| Q2 (0.006-0.018) | | 6,116 | 0.74 (0.61, 0.91) | 0.004 | 0.76 (0.62, 0.93) | 0.008 | 0.73 (0.59, 0.90) | 0.004 | 0.73 (0.59, 0.91) | | 0.005 |
| Q3 (0.018-0.039) | | 6,453 | 1.00 (0.83, 1.19) | 0.964 | 0.96 (0.79, 1.16) | 0.671 | 0.92 (0.75, 1.13) | 0.435 | 0.95 (0.77, 1.17) | | 0.633 |
| Q4 (0.040-0.088) | | 6,418 | 0.80 (0.65, 0.98) | 0.030 | 0.77 (0.62, 0.96) | 0.021 | 0.78 (0.62, 0.98) | 0.033 | 0.79 (0.63, 0.99) | | 0.044 |
| Q5 (≥ 0.089) | | 6,101 | 0.88 (0.70, 1.10) | 0.257 | 0.82 (0.64, 1.03) | 0.095 | 0.82 (0.63, 1.05) | 0.120 | 0.83 (0.64, 1.07) | | 0.159 |
| Trend p | |  | 0.722 |  | 0.320 |  | 0.469 |  | 0.525 | |  |

Abbreviations: Q1 to Q5, quintile 1 to 5; OR, odds ratio; CI, confidence interval; Ref, reference; ALA, α-linolenic acid; DPA, docosapentaenoic acid; ETA, eicosatetraenoic acid; EPA, eicosapentaenoic acid; DHA, docosahexenoic acid.

Crude: unadjusted.

Model 1: adjusted for age + sex + race/ethnicity + education + marital status + PIR.

Model 2: adjusted for model 1 + smoking + alcohol intake + stroke + hypertension + hyperlipidemia + diabetes.

Model 3: adjusted for model 2 + dietary supplements + BMI + HDL-C + TC.
