# Supplementary material for: Association between dietary omega-3 intake and coronary heart disease among American adults: The NHANES, 1999–2018
Source: PLoS One. 2023 Dec 20;18(12):e0294861. doi: 10.1371/journal.pone.0294861 (PMC10732455; doi:10.1371/journal.pone.0294861)
Supplement: S3 Table — (DOCX) [file pone.0294861.s003.docx]

**Table S3. Association between dietary omega-3 intake and CHD after exclusion of energy extremes.**

| **Variable** | **N** | **Crude**  **OR (95%CI)** | **P-value** | **Model 1**  **OR (95%CI)** | **P-value** | **Model 2**  **OR (95%CI)** | **P-value** | **Model 3**  **OR (95%CI)** | **P-value** |
| --- | --- | --- | --- | --- | --- | --- | --- | --- | --- |
| Omega-3 (g/d) | | | | | | | | | |
| Q1 (≤ 1.00) | 5,973 | 1(Ref) |  | 1(Ref) |  | 1(Ref) |  | 1(Ref) |  |
| Q2 (1.01-1.41) | 6,106 | 1.0 (0.8, 1.2) | 0.745 | 0.94 (0.77, 1.15) | 0.560 | 0.95 (0.76, 1.19) | 0.669 | 0.94 (0.75, 1.17) | 0.565 |
| Q3 (1.42-1.87) | 6,166 | 0.9 (0.7, 1.1) | 0.435 | 0.94 (0.76, 1.16) | 0.554 | 0.96 (0.77, 1.20) | 0.706 | 0.93 (0.74, 1.17) | 0.529 |
| Q4 (1.88-2.57) | 6,123 | 0.8 (0.6, 1.0) | 0.040 | 0.78 (0.62, 0.99) | 0.041 | 0.80 (0.63, 1.02) | 0.071 | 0.81 (0.63, 1.03) | 0.094 |
| Q5 (≥ 2.58) | 6,098 | 0.8 (0.6, 1.0) | 0.036 | 0.77 (0.61, 0.97) | 0.028 | 0.81 (0.64, 1.03) | 0.087 | 0.78 (0.61, 0.99) | 0.041 |
| Trend p |  | 0.025 |  | 0.023 |  | 0.069 |  | 0.041 |  |

Abbreviations: Q1 to Q5, quintile 1 to 5; OR, odds ratio; CI, confidence interval; Ref, reference; PIR, poverty income ratio; BMI, body mass index.

Crude: unadjusted.

Model 1: adjusted for age + sex + race/ethnicity + education + marital status + PIR.

Model 2: adjusted for model 1 + smoking + alcohol intake + stroke + hypertension + hyperlipidemia + diabetes.

Model 3: adjusted for model 2 + dietary supplements + BMI + HDL-C + TC.
