# Supplementary material for: Association between dietary omega-3 intake and coronary heart disease among American adults: The NHANES, 1999–2018
Source: PLoS One. 2023 Dec 20;18(12):e0294861. doi: 10.1371/journal.pone.0294861 (PMC10732455; doi:10.1371/journal.pone.0294861)
Supplement: S4 Table — (DOCX) [file pone.0294861.s004.docx]

**Table S4.** **Association between dietary omega-3 intake of each component and CHD after exclusion of energy extremes.**

| **Variables** | | **N** | **Crude**  **OR (95%CI)** | **P-value** | **Model 1**  **OR (95%CI)** | **P-value** | **Model 2**  **OR (95%CI)** | **P-value** | **Model 3**  **OR (95%CI)** | **P-value** |
| --- | --- | --- | --- | --- | --- | --- | --- | --- | --- | --- |
| ALA (g/d) | |  | | | | | | | |  |
| Q1 (≤ 0.82) | | 5,681 | 1(Ref) |  | 1(Ref) |  | 1(Ref) |  | 1(Ref) |  |
| Q2 (0.83-1.18) | | 6,447 | 0.89 (0.74, 1.07) | 0.219 | 0.87 (0.72, 1.06) | 0.178 | 0.86 (0.70, 1.06) | 0.158 | 0.84 (0.68, 1.04) | 0.114 |
| Q3 (1.19-1.58) | | 6,330 | 0.97 (0.80, 1.18) | 0.758 | 0.98 (0.79, 1.21) | 0.840 | 1.02 (0.81, 1.27) | 0.887 | 0.99 (0.79, 1.25) | 0.954 |
| Q4 (1.59-2.19) | | 6,302 | 0.76 (0.62, 0.94) | 0.012 | 0.77 (0.62, 0.97) | 0.027 | 0.78 (0.61, 0.99) | 0.041 | 0.77 (0.61, 0.99) | 0.040 |
| Q5 (≥ 2.20) | | 5,706 | 0.77 (0.61, 0.96) | 0.024 | 0.75 (0.59, 0.96) | 0.026 | 0.79 (0.61, 1.02) | 0.069 | 0.75 (0.57, 0.97) | 0.032 |
| Trend p | |  | 0.017 |  | 0.022 |  | 0.061 |  | 0.031 |  |
| DPA (g/d) | |  | | | | | | | |  |
| Q1 (≤ 0.004) | | 5,800 | 1(Ref) |  | 1(Ref) |  | 1(Ref) |  | 1(Ref) |  |
| Q2 (0.005-0.011) | | 6,332 | 0.78 (0.64, 0.96) | 0.021 | 0.87 (0.71, 1.08) | 0.204 | 0.84 (0.68, 1.04) | 0.114 | 0.79 (0.63, 0.99) | 0.043 |
| Q3 (0.012-0.018) | | 6,195 | 0.78 (0.63, 0.96) | 0.019 | 0.92 (0.74, 1.15) | 0.462 | 0.92 (0.74, 1.14) | 0.444 | 0.86 (0.69, 1.07) | 0.177 |
| Q4 (0.019-0.031) | | 6,242 | 0.70 (0.56, 0.86) | 0.001 | 0.79 (0.63, 0.99) | 0.039 | 0.75 (0.60, 0.94) | 0.014 | 0.69 (0.55, 0.87) | 0.002 |
| Q5 (≥ 0.032) | | 5,897 | 0.64 (0.51, 0.82) | <0.001 | 0.75 (0.58, 0.98) | 0.033 | 0.75 (0.57, 0.98) | 0.035 | 0.70 (0.53, 0.91) | 0.011 |
| Trend p | |  | 0.001 |  | 0.030 |  | 0.035 |  | 0.013 |  |
| ETA (g/d) | |  | | | | | | | |  |
| Q1 (≤ 0.060) | | 5,708 | 1(Ref) |  | 1(Ref) |  | 1(Ref) |  | 1(Ref) |  |
| Q2 (0.061-0.101) | | 6,365 | 0.87 (0.71, 1.06) | 0.174 | 0.90 (0.72, 1.11) | 0.322 | 0.89 (0.71, 1.10) | 0.279 | 0.88 (0.70, 1.10) | 0.253 |
| Q3 (0.102-0.147) | | 6,313 | 0.88 (0.73, 1.06) | 0.172 | 0.95 (0.77, 1.16) | 0.612 | 0.91 (0.74, 1.12) | 0.374 | 0.90 (0.74, 1.11) | 0.341 |
| Q4 (0.148-0.217) | | 6,357 | 0.82 (0.67, 1.01) | 0.061 | 0.83 (0.66, 1.04) | 0.115 | 0.80 (0.63, 1.03) | 0.087 | 0.79 (0.61, 1.02) | 0.072 |
| Q5 (≥ 0.218) | | 5,723 | 0.71 (0.57, 0.90) | 0.004 | 0.74 (0.57, 0.96) | 0.023 | 0.67 (0.52, 0.88) | 0.004 | 0.66 (0.51, 0.87) | 0.003 |
| Trend p | |  | 0.004 |  | 0.015 |  | 0.002 |  | 0.002 |  |
| EPA (g/d) | |  | | | | | | | |  |
| Q1 (≤ 0.003) | | 6,359 | 1(Ref) |  | 1(Ref) |  | 1(Ref) |  | 1(Ref) |  |
| Q2 (0.004-0.006) | | 6,285 | 0.85 (0.70, 1.03) | 0.094 | 0.83 (0.68, 1.01) | 0.069 | 0.80 (0.65, 0.98) | 0.030 | 0.78 (0.64, 0.96) | 0.019 |
| Q3 (0.007-0.011) | | 5,886 | 0.72 (0.58, 0.91) | 0.006 | 0.77 (0.60, 0.98) | 0.034 | 0.77 (0.60, 0.98) | 0.035 | 0.73 (0.57, 0.93) | 0.012 |
| Q4 (0.012-0.029) | | 5,843 | 0.87 (0.67, 1.12) | 0.276 | 0.95 (0.72, 1.24) | 0.688 | 0.93 (0.71, 1.23) | 0.627 | 0.89 (0.68, 1.18) | 0.432 |
| Q5 (≥ 0.030) | | 6,093 | 0.85 (0.70, 1.04) | 0.112 | 0.82 (0.67, 1.00) | 0.051 | 0.85 (0.70, 1.04) | 0.128 | 0.83 (0.68, 1.02) | 0.074 |
| Trend p | |  | 0.766 |  | 0.328 |  | 0.771 |  | 0.717 |  |
| DHA (g/d) | |  | | | | | | | |  |
| Q1 (≤ 0.005) | | 5,909 | 1(Ref) |  | 1(Ref) |  | 1(Ref) |  | 1(Ref) | 1(Ref) |
| Q2 (0.006-0.018) | | 5,965 | 0.75 (0.61, 0.92) | 0.007 | 0.75 (0.61, 0.93) | 0.009 | 0.73 (0.59, 0.90) | 0.005 | 0.73 (0.59, 0.91) | 0.005 |
| Q3 (0.018-0.039) | | 6,312 | 1.00 (0.84, 1.20) | 0.998 | 0.95 (0.79, 1.16) | 0.638 | 0.92 (0.74, 1.13) | 0.419 | 0.95 (0.77, 1.17) | 0.609 |
| Q4 (0.040-0.088) | | 6,293 | 0.81 (0.66, 1.00) | 0.050 | 0.77 (0.62, 0.96) | 0.021 | 0.78 (0.62, 0.98) | 0.033 | 0.79 (0.63, 0.99) | 0.044 |
| Q5 (≥ 0.089) | | 5,987 | 0.90 (0.71, 1.14) | 0.383 | 0.81 (0.64, 1.04) | 0.100 | 0.82 (0.63, 1.07) | 0.143 | 0.84 (0.65, 1.09) | 0.190 |
| Trend p | |  | 0.908 |  | 0.332 |  | 0.531 |  | 0.605 |  |

Abbreviations: Q1 to Q5, quintile 1 to 5; OR, odds ratio; CI, confidence interval; Ref, reference; ALA, α-linolenic acid; DPA, docosapentaenoic acid; ETA, eicosatetraenoic acid; EPA, eicosapentaenoic acid; DHA, docosahexenoic acid.

Crude: unadjusted.

Model 1: adjusted for age + sex + race/ethnicity + education + marital status + PIR.

Model 2: adjusted for model 1 + smoking + alcohol intake + stroke + hypertension + hyperlipidemia + diabetes.

Model 3: adjusted for model 2 + dietary supplements + BMI + HDL-C + TC.
