# Supplementary material for: Association between dietary omega-3 intake and coronary heart disease among American adults: The NHANES, 1999–2018
Source: PLoS One. 2023 Dec 20;18(12):e0294861. doi: 10.1371/journal.pone.0294861 (PMC10732455; doi:10.1371/journal.pone.0294861)
Supplement: S5 Table — (DOCX) [file pone.0294861.s005.docx]

**Table S5.**  **Association between dietary omega-3 intake and CHD after exclusion of BMI extremes.**

| **Variable** | **N** | **Crude**  **OR (95%CI)** | **P-value** | **Model 1**  **OR (95%CI)** | **P-value** | **Model 2**  **OR (95%CI)** | **P-value** | **Model 3**  **OR (95%CI)** | **P-value** |
| --- | --- | --- | --- | --- | --- | --- | --- | --- | --- |
| Omega-3 (g/d) | | | | | | | | | |
| Q1 (≤ 1.00) | 4,072 | 1(Ref) |  | 1(Ref) |  | 1(Ref) |  | 1(Ref) |  |
| Q2 (1.01-1.41) | 4,006 | 0.95 (0.74, 1.23) | 0.721 | 0.93 (0.71, 1.22) | 0.590 | 0.97 (0.72, 1.30) | 0.825 | 0.93 (0.70,1.25) | 0.643 |
| Q3 (1.42-1.87) | 4,098 | 0.85 (0.67, 1.08) | 0.186 | 0.83 (0.65, 1.07) | 0.148 | 0.88 (0.67, 1.15) | 0.354 | 0.84 (0.64,1.11) | 0.217 |
| Q4 (1.88-2.57) | 3,996 | 0.75 (0.58, 0.97) | 0.028 | 0.72 (0.55, 0.95) | 0.021 | 0.79 (0.59, 1.04) | 0.097 | 0.77 (0.58,1.03) | 0.084 |
| Q5 (≥ 2.58) | 3,921 | 0.74 (0.57, 0.95) | 0.019 | 0.71 (0.55, 0.93) | 0.013 | 0.77 (0.58, 1.02) | 0.071 | 0.74 (0.55,0.98) | 0.039 |
| Trend p |  | 0.013 |  | 0.011 |  | 0.057 |  | 0.041 |  |

Abbreviations: BMI, body mass index; Q1 to Q5, quintile 1 to 5; OR, odds ratio; CI, confidence interval; Ref, reference.

Crude: unadjusted.

Model 1: adjusted for age + sex + race/ethnicity + education + marital status + PIR.

Model 2: adjusted for model 1 + smoking + alcohol intake + stroke + hypertension + hyperlipidemia + diabetes.

Model 3: adjusted for model 2 + dietary supplements + BMI + HDL-C + TC.
