# Supplementary material for: Association between dietary omega-3 intake and coronary heart disease among American adults: The NHANES, 1999–2018
Source: PLoS One. 2023 Dec 20;18(12):e0294861. doi: 10.1371/journal.pone.0294861 (PMC10732455; doi:10.1371/journal.pone.0294861)
Supplement: S6 Table — (DOCX) [file pone.0294861.s006.docx]

**Table S6.** **Association between dietary omega-3 component intake and CHD after exclusion of BMI extremes.**

| **Variables** | | **N** | **Crude**  **OR (95%CI)** | **P-value** | **Model 1**  **OR (95%CI)** | **P-value** | **Model 2**  **OR (95%CI)** | **P-value** | **Model 3**  **OR (95%CI)** | | **P-value** |
| --- | --- | --- | --- | --- | --- | --- | --- | --- | --- | --- | --- |
| ALA (g/d) | |  | | | | | | |  |  |  |
| Q1 (≤ 0.82) | | 3,867 | 1(Ref) |  | 1(Ref) |  | 1(Ref) |  | 1(Ref) | |  |
| Q2 (0.83-1.18) | | 4,216 | 0.81 (0.63, 1.03) | 0.087 | 0.78 (0.60, 1.02) | 0.067 | 0.79 (0.59, 1.04) | 0.096 | 0.76 (0.57,1.00) | | 0.054 |
| Q3 (1.19-1.58) | | 4,229 | 0.95 (0.74, 1.22) | 0.695 | 0.93 (0.71, 1.22) | 0.607 | 0.98 (0.73, 1.31) | 0.871 | 0.93 (0.69,1.26) | | 0.648 |
| Q4 (1.59-2.19) | | 4,139 | 0.66 (0.52, 0.83) | 0.001 | 0.65 (0.51, 0.84) | 0.001 | 0.68 (0.52, 0.90) | 0.007 | 0.66 (0.50,0.87) | | 0.004 |
| Q5 (≥ 2.20) | | 3,642 | 0.70 (0.54, 0.92) | 0.011 | 0.70 (0.53, 0.92) | 0.013 | 0.76 (0.56, 1.04) | 0.089 | 0.72 (0.52,0.99) | | 0.048 |
| Trend p | |  | 0.007 |  | 0.009 |  | 0.073 |  | 0.047 | |  |
| DPA (g/d) | |  | | | | | | |  |  |  |
| Q1 (≤ 0.004) | | 4,104 | 1(Ref) |  | 1(Ref) |  | 1(Ref) |  | 1(Ref) | |  |
| Q2 (0.005-0.011) | | 4,255 | 0.76 (0.59, 0.97) | 0.030 | 0.80 (0.63, 1.02) | 0.079 | 0.78 (0.61, 0.99) | 0.042 | 0.71 (0.55,0.91) | | 0.008 |
| Q3 (0.012-0.018) | | 3,985 | 0.67 (0.52, 0.86) | 0.002 | 0.74 (0.57, 0.95) | 0.020 | 0.75 (0.58, 0.97) | 0.031 | 0.67 (0.52,0.88) | | 0.004 |
| Q4 (0.019-0.031) | | 3,924 | 0.69 (0.51, 0.92) | 0.011 | 0.71 (0.53, 0.96) | 0.026 | 0.70 (0.51, 0.96) | 0.028 | 0.63 (0.46,0.86) | | 0.004 |
| Q5 (≥ 0.032) | | 3,825 | 0.60 (0.45, 0.78) | <0.001 | 0.64 (0.48, 0.86) | 0.003 | 0.64 (0.47, 0.86) | 0.004 | 0.58 (0.43,0.79) | | 0.001 |
| Trend p | |  | 0.002 |  | 0.008 |  | 0.011 |  | 0.004 | |  |
| ETA (g/d) | |  | | | | | | |  |  |  |
| Q1 (≤ 0.060) | | 4,098 | 1(Ref) |  | 1(Ref) |  | 1(Ref) |  | 1(Ref) | |  |
| Q2 (0.061-0.101) | | 4,294 | 0.82 (0.65, 1.04) | 0.105 | 0.80 (0.62, 1.03) | 0.087 | 0.78 (0.60, 1.02) | 0.071 | 0.78 (0.60,1.01) | | 0.060 |
| Q3 (0.102-0.147) | | 4,123 | 0.81 (0.63, 1.04) | 0.102 | 0.84 (0.64, 1.09) | 0.192 | 0.82 (0.62, 1.09) | 0.167 | 0.81 (0.61,1.07) | | 0.143 |
| Q4 (0.148-0.217) | | 4,056 | 0.78 (0.60, 1.02) | 0.076 | 0.76 (0.56, 1.04) | 0.092 | 0.73 (0.52, 1.02) | 0.072 | 0.73 (0.52,1.03) | | 0.075 |
| Q5 (≥ 0.218) | | 3,522 | 0.69 (0.53, 0.90) | 0.007 | 0.68 (0.51, 0.91) | 0.010 | 0.63 (0.46, 0.85) | 0.003 | 0.61 (0.45,0.84) | | 0.003 |
| Trend p | |  | 0.012 |  | 0.020 |  | 0.008 |  | 0.006 | |  |
| EPA (g/d) | |  | | | | | | |  |  |  |
| Q1 (≤ 0.003) | | 4,400 | 1(Ref) |  | 1(Ref) |  | 1(Ref) |  | 1(Ref) | |  |
| Q2 (0.004-0.006) | | 4,181 | 0.81 (0.62, 1.06) | 0.130 | 0.77 (0.58, 1.01) | 0.059 | 0.71 (0.54, 0.94) | 0.019 | 0.69 (0.52,0.90) | | 0.008 |
| Q3 (0.007-0.011) | | 3,779 | 0.68 (0.50, 0.93) | 0.018 | 0.67 (0.49, 0.92) | 0.014 | 0.67 (0.48, 0.94) | 0.021 | 0.63 (0.45,0.88) | | 0.007 |
| Q4 (0.012-0.029) | | 3,616 | 0.75 (0.57, 0.98) | 0.039 | 0.79 (0.59, 1.05) | 0.110 | 0.81 (0.59, 1.09) | 0.168 | 0.77 (0.57,1.04) | | 0.089 |
| Q5 (≥ 0.030) | | 4,117 | 0.84 (0.66, 1.07) | 0.168 | 0.76 (0.60, 0.97) | 0.032 | 0.78 (0.60, 1.01) | 0.059 | 0.75 (0.58,0.97) | | 0.030 |
| Trend p | |  | 0.972 |  | 0.415 |  | 0.688 |  | 0.654 | |  |
| DHA (g/d) | |  | | | | | | |  |  |  |
| Q1 (≤ 0.005) | | 4,050 | 1(Ref) |  | 1(Ref) |  | 1(Ref) |  | 1(Ref) | |  |
| Q2 (0.006-0.018) | | 3,930 | 0.69 (0.52, 0.92) | 0.012 | 0.68 (0.50, 0.91) | 0.011 | 0.66 (0.49, 0.89) | 0.007 | 0.66 (0.49,0.89) | | 0.008 |
| Q3 (0.019-0.039) | | 4,120 | 0.95 (0.74, 1.21) | 0.665 | 0.89 (0.69, 1.14) | 0.349 | 0.84 (0.65, 1.09) | 0.188 | 0.87 (0.67,1.13) | | 0.303 |
| Q4 (0.040-0.088) | | 4,007 | 0.82 (0.65, 1.05) | 0.114 | 0.78 (0.61, 1.00) | 0.054 | 0.78 (0.60, 1.01) | 0.060 | 0.79 (0.61,1.02) | | 0.070 |
| Q5 (≥ 0.089) | | 3,986 | 0.86 (0.64, 1.16) | 0.331 | 0.76 (0.56, 1.02) | 0.074 | 0.77 (0.56, 1.05) | 0.106 | 0.80 (0.58,1.11) | | 0.180 |
| Trend p | |  | 0.904 |  | 0.355 |  | 0.519 |  | 0.672 | |  |

Abbreviations: BMI, body mass index; Q1 to Q5, quintile 1 to 5; OR, odds ratio; CI, confidence interval; Ref, reference; ALA, α-linolenic acid; DPA, docosapentaenoic acid; ETA, eicosatetraenoic acid; EPA, eicosapentaenoic acid; DHA, docosahexenoic acid.

Crude: unadjusted.

Model 1: adjusted for age + sex + race/ethnicity + education + marital status + PIR.

Model 2: adjusted for model 1 + smoking + alcohol intake + stroke + hypertension + hyperlipidemia + diabetes.

Model 3: adjusted for model 2 + dietary supplements + BMI + HDL-C + TC.
