# Supplementary material for: Association between dietary omega-3 intake and coronary heart disease among American adults: The NHANES, 1999–2018
Source: PLoS One. 2023 Dec 20;18(12):e0294861. doi: 10.1371/journal.pone.0294861 (PMC10732455; doi:10.1371/journal.pone.0294861)
Supplement: S1 Fig — (DOCX) [file pone.0294861.s007.docx]

**
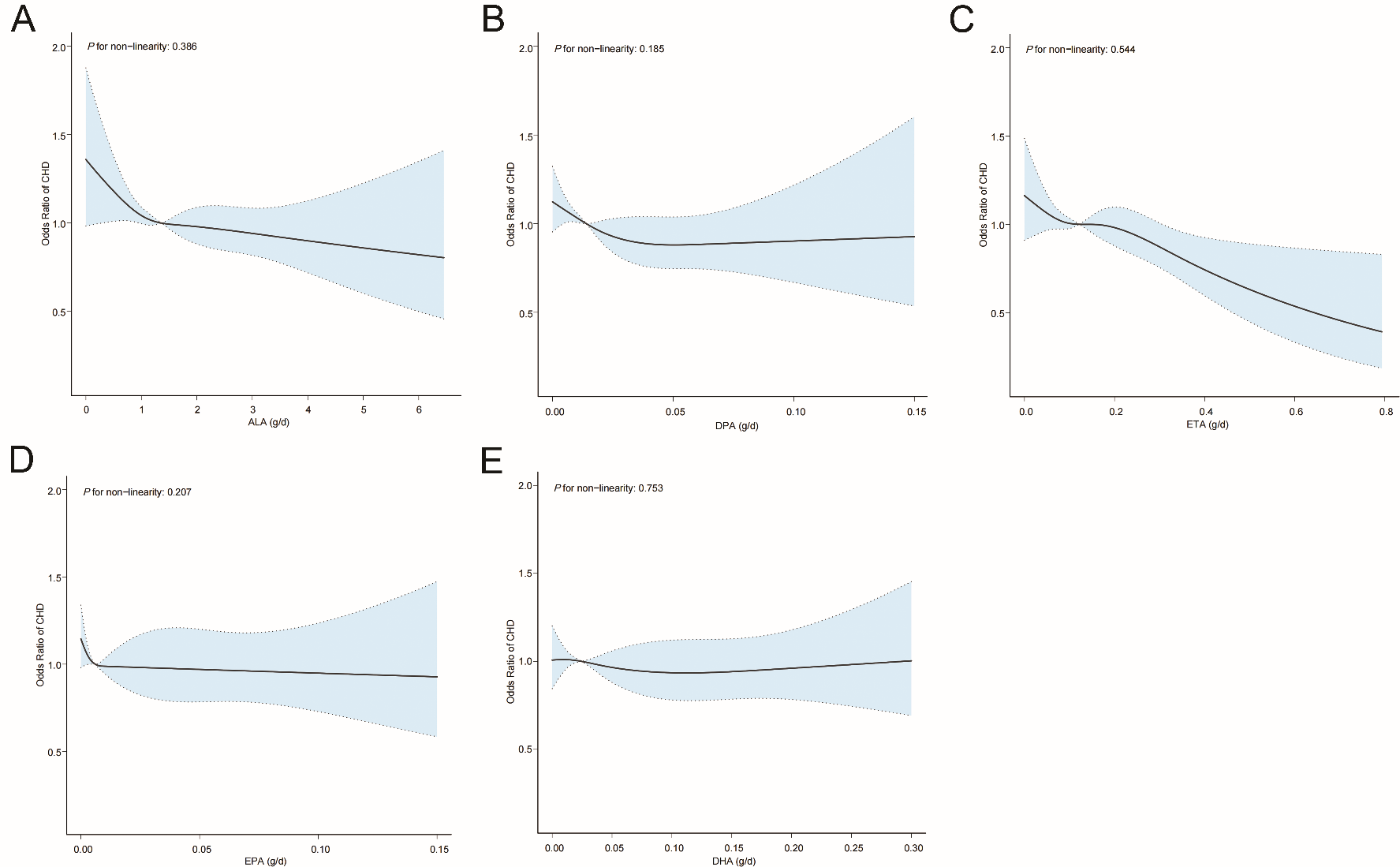
**

**Figure S1. Dose-response relationship between dietary ALA(A), DPA(B), ETA (C), EPA(D), DHA(E) intake and CHD.**

The solid line indicates the estimated risk of CHD, and the dashed line indicates the fitted 95% CI.

Abbreviations: ALA, α-linolenic acid; DPA, docosapentaenoic acid; ETA, eicosatetraenoic acid; EPA, eicosapentaenoic acid; DHA, docosahexenoic acid.

We adjusted for age, sex, race/ethnicity, education, marital status, PIR, smoking, alcohol intake, stroke, hypertension, hyperlipidemia, diabetes, dietary supplements, BMI, HDL-C, TC.
